# Supplementary material for: Farmed oyster mortality follows consistent Vibrio community reorganization
Source: mSystems. 2025 Oct 2;10(10):e01078-25. doi: 10.1128/msystems.01078-25 (PMC12542681; doi:10.1128/msystems.01078-25)
Supplement: Supplemental Material — Figures S1 to S3 and Table S2. [file msystems.01078-25-s0001.docx]

**
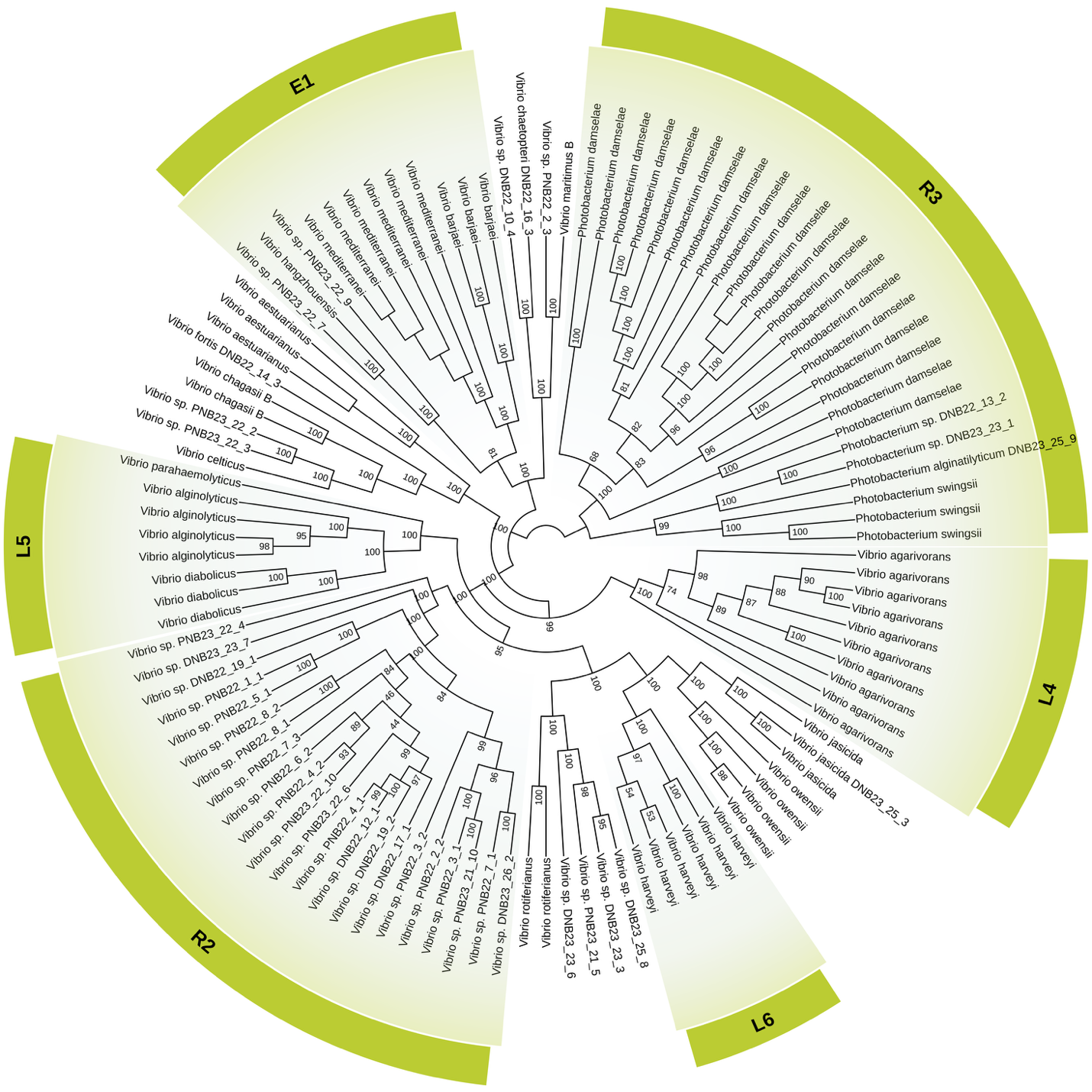
**

**Supplemental Figure S1.** Phylogeny of Vibrios associated with healthy and diseased eastern oysters. Maximum-likelihood phylogenetic tree based on core-genome alignments from 110 Vibrio isolates in this study. Conserved clades described in main text Figure 1 are labeled and highlighted, with individual nodes labeled according to GTDB-Tk-assigned taxonomy. Bootstrap support and approximate likelihood-ratio tests (SH-aLRT) were performed with 1000 replicates each to assess branch support.


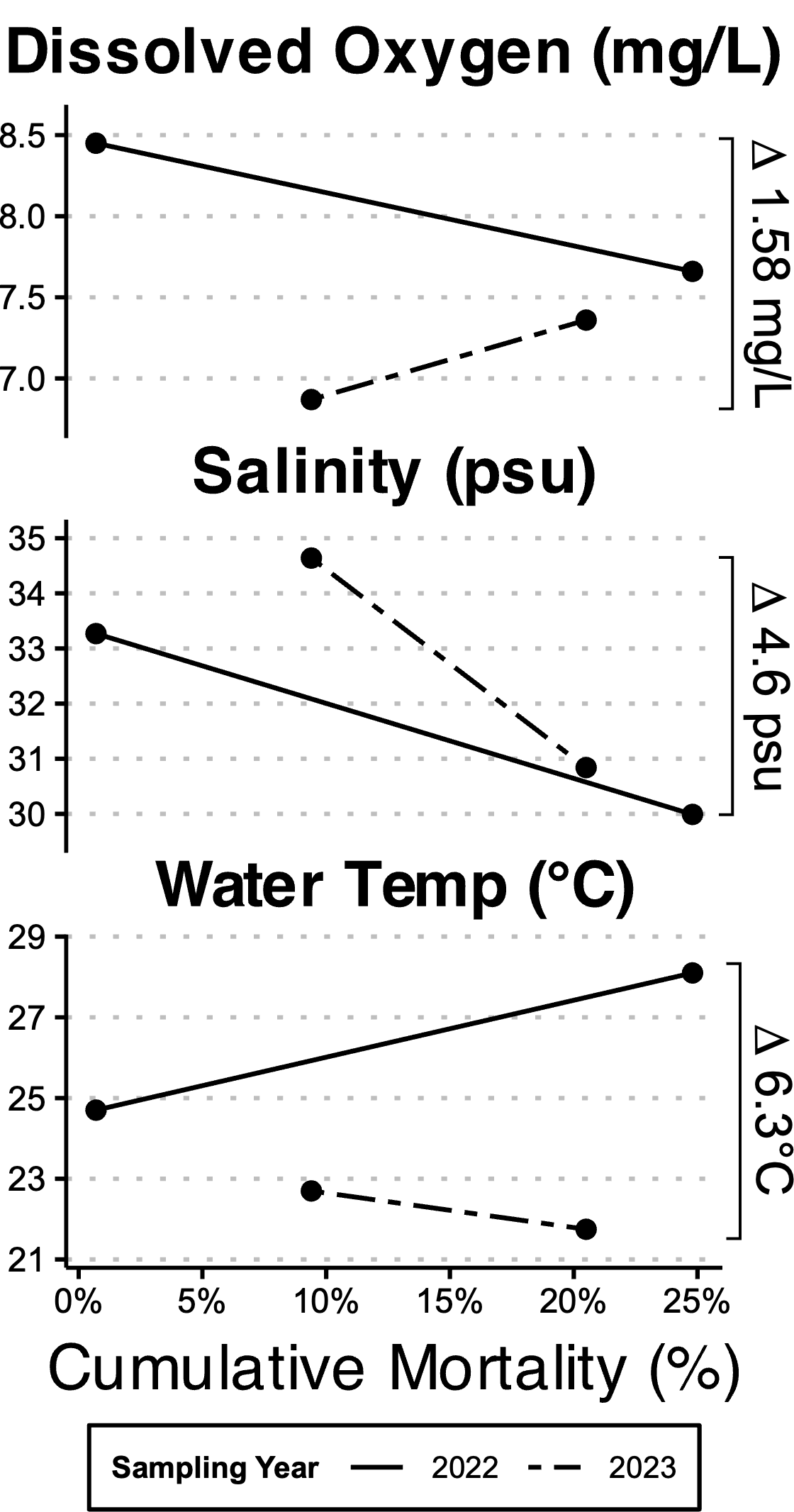


**Supplemental Figure S2.** Environmental Conditions During Sampling Events. Environmental parameters measured by YSI at each sampling event, with percent cumulative mortality at the time of sampling on the x-axis, and range of values indicated on the right y-axis.


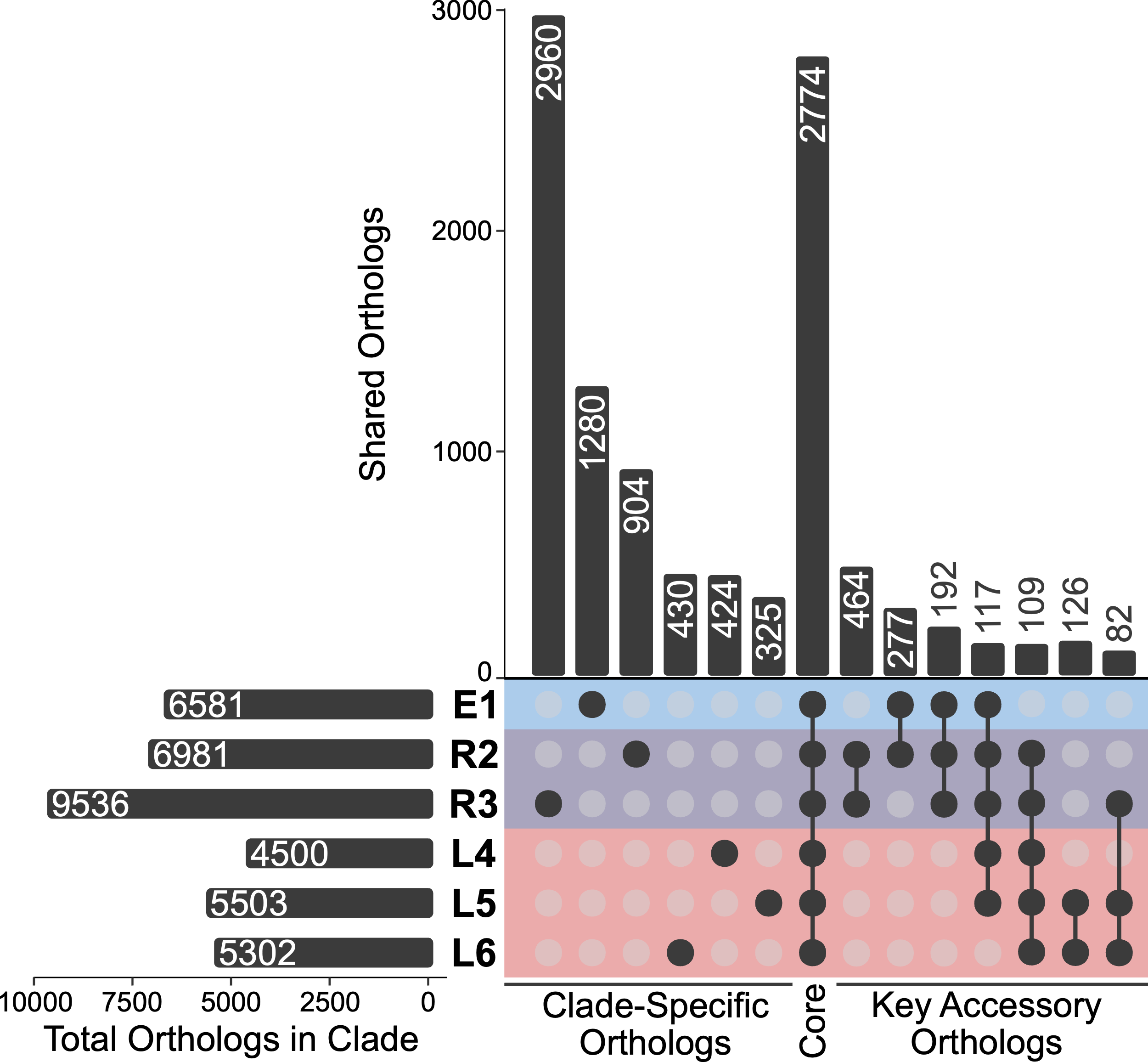


**Supplementary Figure S3.** Core and accessory orthologs across Vibrio clades. (Left) Total number of orthologs identified in each Vibrio clade. (Right) Upset plot showing the number of orthologs unique to each clade (clade-specific), core orthologs, and accessory orthologs shared between key clade pairs. V. mediterranei-specific orthologs are lost from the community, and V. harveyi-specific orthologs are introduced in the transition from initial to ongoing mortality.


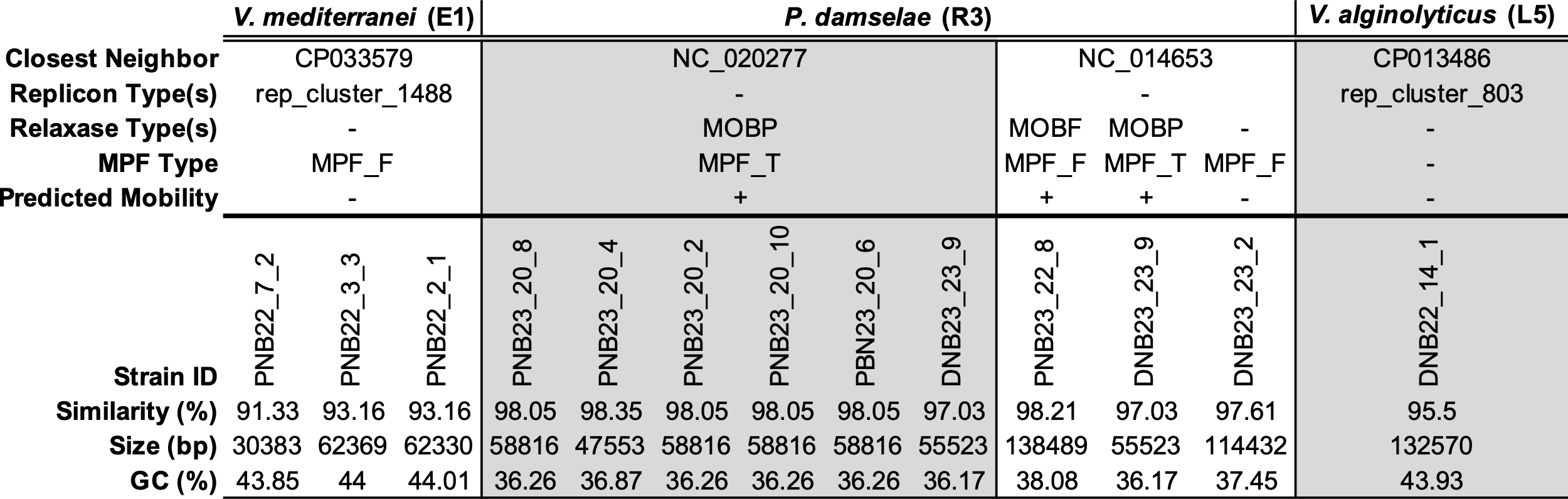


**Supplementary Table S2.** Plasmid distribution among Vibrio clades. Plasmid presence in all strains was evaluated using MOB-suite. Closest neighbor, replicon, relaxase, and MPF type, along with predicted mobility, directly reflect MOB-suite output. Similarity (%) represents the percent identity shared between plasmids identified here and their closest neighbor.
